# The General Nature and Treatment of Tumours

**Published:** 1845-10

**Authors:** 


### THE GENERAL NATURE AND TREATMENT OF TUMOURS. By *George Macilwain*, &c. &c. 8vo. pp. 219. London, Churchill, 1845.

MR. MACILWAIN commences his Preface by stating, "in the following pages I have endeavoured to *sketch* the principles and application of what I call 'Organic Surgery,' to the treatment of tumours." This assertion, and the title of the work, led us to expect some novel views with regard to the anatomy and pathology, as well as the treatment, of the various tumours to which the human frame is so lamentably subject; and we hoped to have met with an improved classification, or at all events an amendment upon the attempts at arrangement already made. We have, however, been disappointed; for, although the book contains many valuable observations, mostly truisms, with some few exceptions, they are equally applicable to all chronic diseases, and the work would have consequently borne a corresponding title with nearly equal truth to the one adopted.

Our author makes frequent allusion to his other published labours, which implies the necessity of consulting them in order to properly understand the work under notice; whether such perusal would be profitably bestowed we have not yet had the opportunity of deciding, but certain it is, our author's allusions shew the good opinion he entertains of their merits.

"Organic Surgery" is hardly a happy term, as not conveying the intended meaning, as it may imply the surgery of the different organs, or of an organic tissue, and we are not quite sure we are correct in understanding it here to refer to the investigation of all the organs of the body in local disease, and the treatment of such as are detected in fault. The apparent objects of the work are, first, to impress the fact that all tumours are consequent upon an interruption of function of one or more of the great secreting and exhaling organs; and that many of them are deposits of chemical combinations which should have been separated from the body by the normal action of a healthy organ, but, being retained, the general system is relieved by the local deposit. And, in the second place, to direct attention to the necessity of carefully investigating the condition of every organ of the body, particularly of course those of vegetative life, to ascertain the structure in fault, to which, being the primary seat of disease, medical remedies are to be applied, and not to the tumour itself, the secondary or consequent affection. Thus carrying out the views, but hardly improving upon them, for which the world is so deeply indebted to the late Mr. Abernethy.

Mr. Macilwain's sweeping theory of the origin of all tumours may fairly be disputed; that many of the mild character arise, as he describes, has been admitted and taught for a long period; but there are many the connection of which with general disorder is very doubtful; and others which plainly have no reference to disease of the organs of nutrition; in the first class we allude to tumours evidently excited by mechanical injury, or occurring in a structure about to assume or part with a function, as in the breast, uterus, or testicle, in many of which cases the inference is rather that the local disease is primary and the general affection, if any, secondary; in the second class we would refer to tumours formed by parasitic animals; although we willingly admit the present uncertainty of their origin in many instances, and incline to the belief that a general deficiency of organism is occasionally connected with, though most likely not giving rise to, their formation and growth, yet we have no doubt that the majority of such cases are entirely local, with the formation of which the general health has nothing to do, however it may be influenced by the progress of the tumour.

Among much heterogeneous matter, and we feel unwillingly obliged to add, unsuccessful attempts at "philosophical induction," Mr. Macilwain's work contains many useful "hints" for the improvement of practice in these and other chronic diseases, and the more perfectly to carry the practice into operation, he has recommended a "Table of Record" of the previous and existing condition of the patient, of the inherent and surrounding circumstances, &c.; similar in many particulars to those used by the clinical clerks of our own hospitals, as well as on the Continent.* We entirely coincide in the author's opinion of the usefulness, nay in many instances of the necessity, of such minute examination, and of recording the altered conditions from time to time; but it is equally certain that it can admit only of partial application; in the routine of practice, neither the physician nor the surgeon has time to devote to such record, even if the patient would submit to this careful examination, excepting in cases of urgent seriousness and great interest; at the same time, it would be very advantageous to the public health and to professional usefulness, if every student in medicine and young practitioner were to habituate himself to some such investigation and record of the condition of every organ of the body, his mind being thus schooled, would more easily and rapidly take in the leading features of the health or disease of individuals in his after more busy life, a tact which will assuredly be of infinite value both to himself and his patients.

Mr. Macilwain seems to possess great confidence in the cure of tumours not positively malignant, through the restoration of the general health, or the health of any one or more disturbed organs, and not a little in the amelioration of incurable diseases; and anticipates the time when, by the further advance of the sciences of disease and of chemistry, even those now justly regarded as malignant, will yield to the appropriate treatment. We sincerely hope his anticipations will be realised, though at present the desideratum appears to be far distant.

We are presented with some good observations under the head "Of Health and Disease, and of Conditions of Transition from one to the other," and regret that they are so mingled with matter holding little or no connection with the subject. We have long been impressed with the fact of

* The author notices that he was the first who delivered clinical lectures in the Metropolis, a circumstance especially creditable, and the more so, if it were that example which has led to their general introduction in the practice of medical tuition.

disease existing without giving rise to symptoms, or if they exist are only to be detected by more than ordinary careful investigation. Mr. Macilwain says, "I hear continually both patients and medical men say, 'the general health is good,' 'there seems nothing wrong in the general health,' 'all the functions seem properly performed,' &c. in cases where nothing is more easy than to demonstrate the contrary. Now, in morbid anatomy, an important truly, but still a comparatively very small, branch of enquiry, no man thinks himself competent even to study, much less to apply it, without informing himself thoroughly as to the marks which distinguish healthy structure; and if a system of surgery is to be recommended which is professedly based, no matter what the disease is, on correcting the functions of various organs which may be faulty, the whole matter turns on our distinguishing what is faulty and what is not so." "Now the determination of the signs of health are not so simple as many persons may imagine." In allusion to "compensating functions" we have this observation, "The very first fact we perceive, when disease, *commonly* so called, does occur, is, that it is not seated in the organ to whose functions the disturbing causes were originally applied, but to that which had been employed in the *compensating function.*" Mr. Macilwain modifies, properly enough, this important assertion further on, which is of frequent application but by no means universal.

Probably the most valuable portion of this work is contained in the "Hints on the Management of different Organs;" Mr. Macilwain recommends an accurate register of diet to be kept in a "Diet-book," to afford both patient and practitioner an opportunity of ascertaining and correcting what may be wrong: perhaps the most annoying and troublesome part of the treatment of chronic diseases exists in the regulation of diet, and therefore any assistance, however slight, will be valuable. We are gratified in reading many useful "hints" in the general regulation of the nutrient organs, and regret that, among much practical matter, there appears to us to be mixed up much of error in the physiology in which our author has indulged; he has adopted the idea at the suggestion of "Mr. Maugham, a man of striking talent, and a bold and original thinker," that the lungs are merely "refrigerating organs," and supported it by arguments which may be easily refuted by the tyro in physiology; that the lungs are "refrigerating organs" we have no doubt, but that they serve also higher and more essential purposes in the animal economy may be readily proved. Mr. Macilwain, however, "hopes to find time to publish, in a complete form, the whole facts on which the allegations are founded;" until we have then an opportunity of correcting our opinions we must consider that our author would have better consulted his own reputation, and the usefulness of his book, if he had confined it to the really good practical matter it contains, rather than have attempted to shew himself a philosopher, in which he has signally failed. Wisely enough, little dependence is placed on local measures to tumours, yet they are not altogether so valueless as represented. Mr. Macilwain says, "there is no such thing, in my opinion, as a 'local application' in surgery, if by that term is meant a measure whose influence is necessarily confined to the part."

The author has refused to the profession in general the credit it deserves, not doing it justice corresponding to its acquirements, and some authors and teachers he has certainly misrepresented, circumstances attributable to a feeling, which is clearly evidenced in his work, of the superiority of his own mind and information,—a feeling which leads him to assume the tone of philosophical dictation, for which his present acquirements do not fit him—to this same feeling may be attributed the colloquial and often careless style in the language used, which sometimes almost excites a smile. Notwithstanding, we recommend the careful perusal of the work to the whole profession, but especially to its younger members, as the wheat which it contains may readily be separated from the chaff.

